# Enhanced Acetone-Sensing Performance of a Bilayer Structure Gas Sensor Composed of a ZnO Nanorod Top Layer and a ZnFe_2_O_4_ Nanoparticle Decorated ZnO Nanorod Bottom Layer

**DOI:** 10.3390/s24237851

**Published:** 2024-12-08

**Authors:** Hao Wu, Huichao Zhu, Jianwei Zhang, Jun Yu, Zhenan Tang, Guanyu Yao, Wenqing Zhao, Guohui Wu, Xia Jin

**Affiliations:** 1 School of Control Science and Engineering, Dalian University of Technology, Dalian 116024, China; 2 Key Lab of Liaoning for Integrated Circuits and Medical Electronic Systems, Dalian University of Technology, Dalian 116024, China; 3 School of Biomedical Engineering, Dalian University of Technology, Dalian 116024, China; 4 Department of Biomedical Engineering, School of Intelligent Medicine, China Medical University, Shenyang 110122, China

**Keywords:** ZnFe_2_O_4_ nanoparticle-decorated ZnO nanorod, bilayer structure, acetone sensor, heterojunction

## Abstract

In this study, we report a high-performance acetone gas sensor utilizing a bilayer structure composed of a ZnO nanorod top layer and a ZnFe_2_O_4_ nanoparticle-decorated ZnO nanorod bottom layer. ZnO nanorods were synthesized via a water-bath method, after which the ZnFe_2_O_4_ nanoparticle-decorated ZnO nanorods were prepared using a simple immersion and calcination method. SEM and TEM revealed the porous morphology of the samples and the formation of ZnO-ZnFe_2_O_4_ heterojunctions. XPS analysis demonstrated an increase in oxygen vacancy content with the introduction of ZnFe_2_O_4_ nanoparticles. Compared to pure ZnO nanorods, ZnFe_2_O_4_-decorated ZnO nanorods showed a 3.9-fold increase in response to 50 ppm acetone. Covering this layer with ZnO nanorods further increased the response by an additional 1.6 times, and simultaneously enhanced the selectivity to acetone. The top layer improves gas sensing performance by introducing heterojunctions with the bottom layer, partially blocking acetone gas at the bottom layer to facilitate a more complete reaction, and filtering ethanol interference.

## 1. Introduction

Acetone (C_3_H_6_O) is a colorless, transparent organic liquid with a characteristic sweet odor. It is widely used in various industries, including chemical production, pesticides, pharmaceuticals, construction, and military, serving as a versatile reagent such as solvent, cleaning agent, diluent, and extraction agent [1,2,3,4,5]. Acetone has high volatility since its saturated vapor pressure calculated from the Antoine Equation is ~4.2 times higher than that of ethanol at 25 °C. Due to its high volatility, acetone vapor can rapidly disperse into the air. Acetone vapor is highly flammable and can form explosive mixtures with air at concentrations between 2.2% and 14% [6]. Prolonged or high-level exposure to acetone vapor can result in damage to the nervous system, respiratory system, and eyes, causing symptoms like dizziness, headaches, nausea, and slow nerve response [1,7]. In China, according to the standard “Occupational exposure limits for hazardous agents in the workplace” (GBZ2.1-2019), the time-weighted average (TWA) concentration and short-term exposure limit (STEL) of acetone vapor are 300 mg/m^3^ (126 ppm) and 500 mg/m^3^ (190 ppm), respectively. In breath testing, acetone is also a marker for determining diabetes, with a detection limit of 1.7 ppm [8,9]. Therefore, there is a great need for accurate and rapid detection of acetone.

Semiconductor gas sensors have shown excellent characteristics such as fast response, high sensitivity, low cost, and ease of integration and manufacture, so they have been widely studied and used in acetone detection [10]. In the sensors, advanced semiconductor materials such as SnO_2_ [11], ZnO [12], Fe_2_O_3_ [13], ZnFe_2_O_4_ [14], etc., are normally fabricated on a ceramic or silicon-based heater [15,16]. Under heating, acetone reacts with adsorbed oxygen on the surface of the material, causing a change in the material’s resistance. The composition and structure of gas-sensing materials are key factors that determine gas-sensing performance since they can significantly affect the response process.

ZnO, ZnFe_2_O_4_, and their composites have emerged as highly promising materials for acetone gas sensing applications. For instance, Du et al. developed ZnO nanofiber-based gas sensors using an electrospinning technique, achieving significant improvements in acetone sensing performance after oxygen plasma treatment. This treatment enhanced the specific surface area and created oxygen-terminated ZnO surfaces, which facilitated acetone adsorption [17]. Similarly, Wen et al. synthesized porous ZnFe_2_O_4_ nanosheets with a 3D reduced graphene oxide template, demonstrating superior acetone sensing performance compared to ZnO and Fe_2_O_3_ nanosheets [18]. Notably, composite materials with well-designed structures and optimized heterojunctions often exhibit enhanced gas-sensing properties. Meng et al. fabricated hollow, urchin-like core-shell ZnO/ZnFe_2_O_4_ spheres, achieving a high response of 94 to 100 ppm acetone (4 times higher than ZnFe_2_O_4_), with response/recovery times of 56/89 s and an initial resistance of around 800 MΩ [19]. Song et al. enhanced acetone sensing by in situ growing ZnFe_2_O_4_ nanoparticles on ZIF-8-derived rhombic dodecahedral ZnO, showing a high response of 225 to 100 ppm acetone (43 times higher than ZnO), with response/recovery times of 6/158 s, and an initial resistance of 2.8 GΩ [20]. In another study, Li et al. enhanced acetone sensing by sequentially modifying ZnO nanomeshes with ZnFe_2_O_4_ nanosheets and Au nanoparticles, exhibiting a response of 30.3 (3 and 5.5 times higher than ZnO- ZnFe_2_O_4_ and ZnO, respectively), with response/recovery times of 1/58 s, and an initial resistance of about 300 MΩ [21]. The above sensors exhibited good gas sensing performance due to their unique morphology design and the n–n heterojunction between ZnO and ZnFe_2_O_4_. However, their initial resistance was quite high, making them difficult to measure in practical applications. Their recovery times were also relatively slow. These materials have hierarchical structures consisting of attached nanoparticles, in which the grain boundaries may increase the resistance and slow down the diffusion of gases. To date, less attention has been given to ZnFe_2_O_4_ nanoparticle-decorated single crystalline ZnO nanorods for acetone sensing. This material has shown excellent catalytic performance in photocatalytic studies [22], and the catalytic activity is also a desirable property for gas sensors. The use of single crystalline ZnO is expected to decrease the resistance to a reasonable range and mitigate the slow diffusion of gases due to the widely distributed grain boundaries among nanoparticles. Overall, the exploration of corresponding preparation conditions and the assessment of its potential applications in gas sensing is of significant importance.

Furthermore, bilayer gas sensors have demonstrated significant potential for enhanced gas-sensing performance. This type of gas sensor requires a rational design of material combinations and optimized morphology. Some bilayer sensors based on materials like SnO_2_/ZnSnO_3_ [23], CeO_2_/Rh-SnO_2_ [24], and SnO_2_/WO_3_ [25] have been successfully developed by now, but those employing ZnFe_2_O_4_/ZnO nanocomposites remain rarely explored.

In this study, sea-urchin-like ZnO nanorods were synthesized, followed by the in-situ growth of ZnFe_2_O_4_ nanoparticles on their surfaces. The microstructure, morphology, and composition of the synthesized materials were characterized using XRD, SEM, TEM, EDS, and XPS. Both single-layer and bilayer gas sensors were fabricated based on these materials. The bilayer sensor consists of a ZnO nanorod top layer and a ZnFe_2_O_4_ nanoparticle-decorated ZnO nanorod bottom layer. Gas-sensing tests revealed that pure ZnO exhibited higher sensitivity to ethanol, while ZnFe_2_O_4_ decoration significantly enhanced the response to acetone. Furthermore, the bilayer sensor demonstrated improved response and selectivity toward acetone compared to the single-layer sensors. The sensing mechanisms of ZnFe_2_O_4_ nanoparticle decoration and the bilayer structure for acetone detection were analyzed in detail.

## 2. Experimental Details

### 2.1. Synthesis of ZnO Nanorods

ZnO nanorods were synthesized using a mild water-bath method. Specifically, 0.1 M zinc nitrate (Zn(NO_3_)_2_) and 1 M sodium hydroxide (NaOH) were dissolved in purified water to form a white suspension. This suspension was then transferred to a glass conical flask fitted with an airtight silicone stopper and heated at 90 °C in a water bath for 2 h, with continuous vigorous stirring to promote the growth of ZnO nanorods. Following the reaction, the white precipitate (ZnO nanorods) was separated by centrifugation at 4000 rpm and washed 3 to 5 times with purified water until the pH of the washing solution reached neutral.

### 2.2. Synthesis of ZnFe_2_O_4_ Nanoparticle Decorated ZnO Nanorods

**Figure 1:**
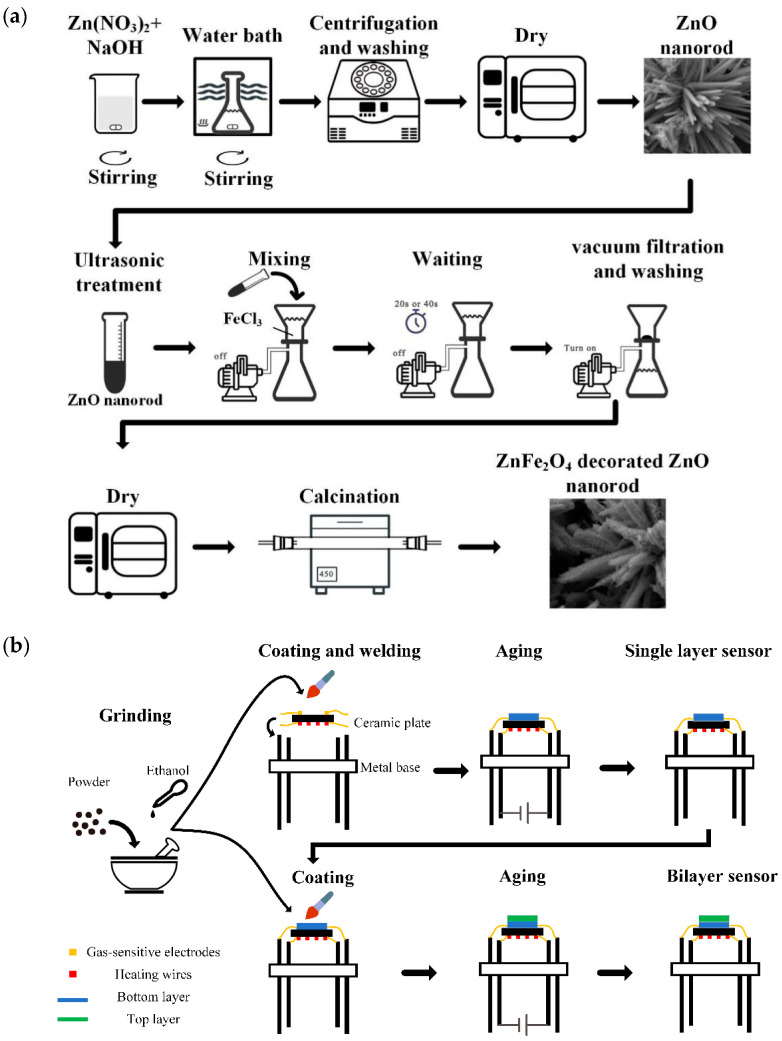
Schematic diagram of (**a**) the synthesis process and (**b**) the sensor fabrication process.

The surface modification of ZnO nanorods with ZnFe_2_O_4_ nanoparticles was accomplished through a straightforward immersion and calcination process. Specifically, 200 mg ZnO nanorods were dispersed in 10 mL purified water ultrasonically. Next, 90 mL 0.01 M FeCl_3_ solution was added to a vacuum filtration apparatus, and the dispersed ZnO nanorods were subsequently introduced into the FeCl_3_ solution. After specific immersion times (20 s and 40 s), the vacuum pump was activated to rapidly filter the FeCl_3_ solution through a 0.45 μm filter membrane (JINTENG, Tianjin, China), leaving the reddish-brown solid retained on the membrane. The collected solid was then washed with purified water, dried in an oven at 90 °C for 5 h, and subsequently calcined in a tubular furnace at 450 °C for 1 h. Ultimately, the ZnFe_2_O_4_ nanoparticle-modified ZnO nanorods were obtained. The samples in this study are differentiated by their immersion times and designated as ZF0, ZF20, and ZF40, where ZF0 represents the ZnO nanorod material that was not immersed in the FeCl_3_ solution. The schematic diagram of the synthesis process is shown in Figure 1a.

### 2.3. Characterization Methods

The structural properties of the samples were characterized by powder X-ray diffraction (XRD, Rigaku SmartLab SE, Rigaku Corp., Tokyo, Japan. Cu Kα radiation, λ = 0.15418 nm) at a scan rate of 2°/min to confirm the presence of ZnO and ZnFe_2_O_4_ phases. The morphology and distribution of ZnFe_2_O_4_ nanoparticles on the ZnO nanorods were examined using a field emission scanning electron microscope (FESEM, JSM-7900F, JEOL, Tokyo, Japan). A high-resolution transmission electron microscope (HRTEM, JEOL JEM-2100F, JEOL, Tokyo, Japan) equipped with an energy-dispersive X-ray spectrometer (EDS) was employed to analyze the crystallinity, internal structure, and elemental distribution. X-ray photoelectron spectroscopy (XPS, Thermo Scientific K-Alpha, Thermo Fisher Scientific Inc., Waltham, MA, USA) was used to investigate the surface composition and elemental states.

### 2.4. Fabrication and Measurement of Gas Sensors

The powdered gas-sensitive material was placed in a mortar, and a few drops of anhydrous ethanol were added. The mixture was rapidly ground to form a stable slurry. Using a fine brush, the slurry was uniformly applied to the surface of an alumina ceramic plate (2 mm × 2 mm× 0.25 mm). Gold gas-sensitive electrodes were fabricated on the front side of the ceramic plate to facilitate resistance measurements of the gas-sensitive material, while a serpentine heating wire was embedded on the back. The sensor, coated with the slurry, was mounted onto a metal base and heated to 300 °C by applying a voltage to the serpentine heating wire, followed by an aging process of 24 h. These single-layer sensors were designated according to their respective materials: ZF0, ZF20, and ZF40. In addition, we also fabricated devices with a bilayer structure. For these, a diluted ZnO nanorod slurry was applied onto the surface of the aged ZF20 and ZF 40 sensors, followed by a secondary aging process. The bilayer sensors were labeled as ZF20-ZF0 and ZF40-ZF0, reflecting the combination of the materials used in their construction. The schematic diagram of the sensor fabrication process is shown in Figure 1b.

The gas-sensing performance of the sensors was evaluated using a static test method. Sensors were placed inside a 48 L transparent cubic chamber with internal dimensions of 40 cm× 30 cm× 40 cm. The sensors’ pins were connected to an external voltage source and a signal collection module via insulated wires. The voltage source powered the serpentine heating wire, while the signal collection module recorded data from multiple sensors. A target gas with a specific concentration was first prepared and stored in a gas bag. Then, a specified volume of target gas was introduced into the test chamber using a syringe, and a fan was employed to ensure rapid and uniform distribution of the gas throughout the chamber. The concentration of the target gas (*C*) in the test chamber can be expressed as *C* = (*V*_s_ × *C*_s_)/*V*_c_, where *V*_s_ is the volume of gas injected using the syringe, *C*_s_ is the concentration of the target gas in the gas bag, and *V*_c_ is the volume of the test chamber (48 L) [26]. After exposure, the chamber was opened to allow the gas to disperse, enabling the sensor signal to return to its baseline state.

The sensor response (*S*) was defined as the ratio of the resistance in air (*R*_a_) to the resistance in the target gas (*R*_g_), i.e., *S* = *R*_a_/*R*_g_. The response and recovery times were defined as the time required for the sensor to exhibit 90% of the response change during the response and recovery phases, respectively. A thermometer and hygrometer were also placed inside the chamber to monitor real-time temperature and humidity conditions during testing. Throughout the testing process, the ambient temperature was maintained at approximately 25 °C. Except for the humidity influence test, the relative humidity is around 30% RH.

## 3. Results and Discussion

### 3.1. Characterization

**Figure 2:**
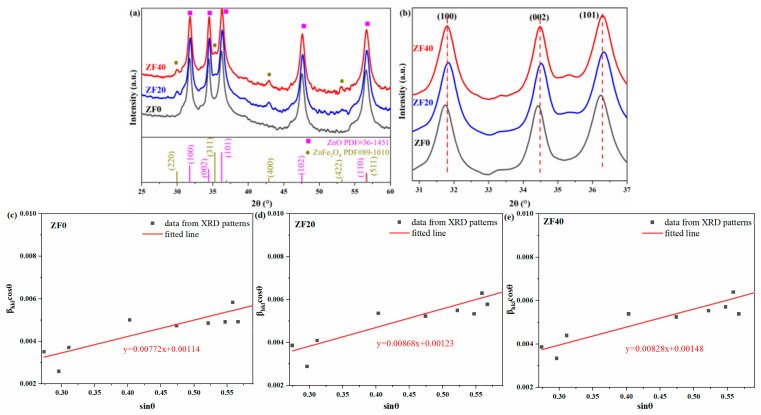
(**a**) XRD patterns, (**b**) magnified patterns, and (**c**–**e**) size-strain plots of ZF0, ZF20, and ZF40.

The crystal structure of the prepared samples was analyzed using X-ray diffraction (XRD), as shown in Figure 2a. Distinct diffraction peaks are observed at approximately 31.74°, 34.40°, 36.22°, 47.52°, and 56.56°, corresponding to the (100), (002), (101), (102), and (110) planes of hexagonal ZnO (PDF#36-1451), respectively. No impurity peaks are detected in the diffraction pattern of the ZF0 sample, indicating that it consists of pure ZnO. In the ZF20 and ZF40 samples, additional low-intensity diffraction peaks appear around 30.06°, 35.31°, 42.96°, and 53.18°, corresponding to the (220), (311), (400), and (422) planes of cubic ZnFe_2_O_4_ (PDF#89-1010). This indicates that these samples comprise a primary ZnO phase and a secondary dopant phase of ZnFe_2_O_4_. The ZnFe_2_O_4_ diffraction peaks are more pronounced in ZF40, suggesting a higher ZnFe_2_O_4_ content in this sample.

The formation of ZnFe_2_O_4_ involves a series of processes, which have been analyzed in studies where ZnO nanorod arrays serve as templates for the preparation of ZnFe_2_O_4_ and Fe_2_O_3_ nanotubes [27,28]. Initially, FeCl_3_ undergoes hydrolysis in solution (Equation (1)), and the resulting H^+^ ions etch the ZnO surface (Equation (2)), leading to the deposition of Fe(OH)_3_ onto the ZnO. As Fe(OH)_3_ is porous and not dense, the H^+^ ions can continue reacting with ZnO through the gaps between Fe(OH)_3_ layers, meaning that the thickness of the Fe(OH)_3_ layer is dependent on the immersion time. Following immersion, during the calcination process, Fe(OH)_3_ dehydrates to form FeOOH, and then Fe_2_O_3_ (Equations (3) and (4)) [29], which then reacts with ZnO to form ZnFe_2_O_4_ (Equation (5)). During this process, Zn atoms from ZnO and Fe atoms from Fe_2_O_3_ diffuse into each other’s lattices, leading to the formation of ZnFe_2_O_4_.

(1) $$Fe^{3+}+3H_{2}O=\mathrm{Fe}{\left(\mathrm{OH}\right)}_{3}+3H^{+}$$

(2) $$\mathrm{ZnO}+2H^{+}={\mathrm{Zn}}^{2+}+H_{2}O$$

(3) $${\mathrm{Fe}(\mathrm{OH})}_{3}=\mathrm{FeOOH}+H_{2}O$$

(4) $$2\mathrm{FeOOH}=Fe_{2}O_{3}+H_{2}O$$

(5) $$ZnO+Fe_{2}O_{3}=\mathrm{ZnF}e_{2}O_{4}$$

Figure 2b shows an enlarged view of the (100), (002), and (101) diffraction peaks of ZnO. It is evident that the diffraction peaks of ZF20 and ZF40 are shifted to higher angles, with shifts of approximately 0.08°–0.1° for ZF20 and 0.06° for ZF40. An increase in the diffraction angle indicates a decrease in the lattice spacing, which may be attributed to Fe^3+^ ion substitution doping in the ZnO lattice and the presence of oxygen vacancies. During the formation of ZnFe_2_O_4_, Fe atoms diffuse into the ZnO lattice, facilitating the transition from the ZnO phase to the ZnFe_2_O_4_ phase. A small portion of Fe atoms may diffuse over long distances, departing from the region where the ZnFe_2_O_4_ phase forms. Their limited number is insufficient to trigger the formation of a new ZnFe_2_O_4_ phase. Consequently, these Fe atoms may occupy Zn vacancies generated by the diffusion of Zn atoms, resulting in substitutional doping. The ionic radii of Fe^3+^ and Zn^2+^ are 0.64 Å and 0.74 Å [30], respectively, so the doping of Fe^3+^ leads to a decrease in lattice spacing. On the other hand, oxygen vacancies may also contribute to the reduction in lattice spacing. DFT calculations have shown that oxygen vacancies with 1+ or 2+ charge state cause lattice expansion, whereas neutral oxygen vacancies induce lattice contraction [31]. After the formation of ZnFe_2_O_4_, a heterojunction is formed with ZnO, and electrons transfer from ZnFe_2_O_4_ to ZnO, facilitating the formation of neutral oxygen vacancies within ZnO, which in turn reduces the lattice spacing. Experimental studies have also reported that oxygen vacancies lead to a reduction in lattice constants [32].

Table 1 presents the lattice parameters and strains of ZnO in various samples, as derived from XRD patterns. These calculations were performed using Jade software. Strain was calculated using the Williamson-Hall method [33,34]. The calculation formula is shown below. The *β_hkl_* and *β_i_* represent the peak broadening of the sample and the instrument, respectively, and *β* is the full width at half maximum (FWHM) of the XRD diffraction peak. *θ*, *D*, λ, and *ε* denote the diffraction angle, average crystallite size, X-ray wavelength (λ = 0.154056 nm), and strain, respectively. A size-strain plot is then constructed, where the y-axis is *β_hkl_* cos*θ* and the x-axis is sin*θ*. Linear fitting is performed on the data points, and from Equation (7), the slope of the fitted line is 4*ε*. The size-strain plots of ZF0, ZF20, and ZF40 are shown in Figure 2c–e.

(6) $$\beta_{hkl}=\sqrt{\beta^{2}-\beta_{i}^{2}}$$

(7) $$\beta_{hkl}\cos\theta =\frac{0.9\lambda }{D}+4\varepsilon \sin\theta$$

As shown in Table 1, both ZF20 and ZF40 exhibit a decrease in lattice parameters, which correlates with the variation in the (101) plane spacing. The lattice parameter of the ZF20 sample shows the greatest reduction, accompanied by the highest strain. This is likely due to the substantial presence of oxygen vacancies within the material, as confirmed by the XPS analysis below.

**Figure 3:**
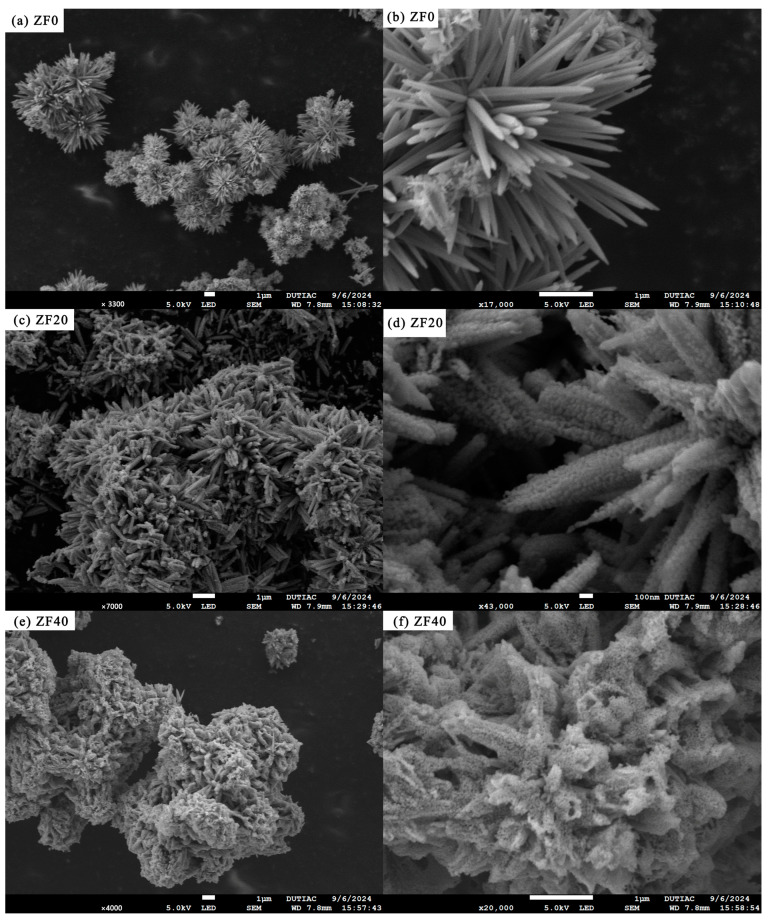
Low and high magnification SEM images of (**a**,**b**) ZF0, (**c**,**d**) ZF20, and (**e**,**f**) ZF40.

Figure 3 presents the SEM images depicting the morphology of various samples. Figure 3a,b reveal that ZnO nanorods are synthesized through a water bath reaction, and under the influence of stirring, these nanorods assemble into an urchin-like spherical structure. This urchin configuration minimizes the dense stacking and aggregation of the nanorods, promoting the rapid diffusion and adsorption of gas molecules within the material, which is advantageous for the sensor’s gas-sensing performance. The nanorods are elongated, straight, and smooth, with an average diameter of 157 nm and an average length of 1.5 µm. Their tips exhibit a needle-like shape, likely a result of corrosion caused by the highly alkaline reaction medium. Figure 3c,d illustrate the morphology of ZF20. After immersion in a 0.01 M FeCl_3_ solution for 20 s followed by calcination at 450 °C, the ZnO nanorod surfaces are coated with ZnFe_2_O_4_ nanoparticles. Numerous ZnFe_2_O_4_-ZnO heterojunctions are also formed on the nanorod surfaces. Additionally, the growth of ZnFe_2_O_4_ nanoparticles causes a slight change in the alignment of the nanorods within the urchin-like structure, leading to a less ordered and more dispersed arrangement compared to ZF0, though a distinct porous structure is still preserved. Figure 3e,f depict the morphology of ZF40. Following immersion in the FeCl_3_ solution for 40 s and subsequent calcination, the ZnFe_2_O_4_ nanoparticles form a thick layer that encapsulates the ZnO nanorods and fills much of the space within the urchin-like structure, drastically altering its morphology. Despite this, the material surface still remains porous. However, the substantial ZnFe_2_O_4_ coating limits the interaction of gas molecules with the ZnFe_2_O_4_-ZnO heterojunction.

**Figure 4:**
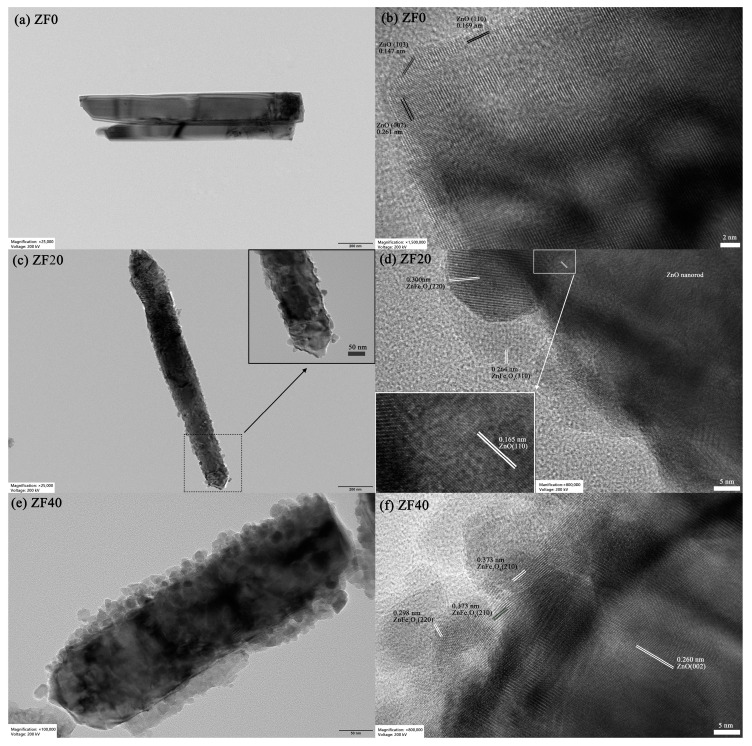
TEM and HRTEM images of (**a**,**b**) ZF0, (**c**,**d**) ZF20, and (**e**,**f**) ZF40.

To gain deeper insights into the morphology and lattice structure of the synthesized samples, TEM and HRTEM images are presented in Figure 4. Figure 4a,c,e depict the microstructures of ZF0, ZF20, and ZF40, respectively. Consistent with the SEM results, the ZF0 sample exhibits a smooth-surfaced nanorod morphology. In contrast, the ZF20 and ZF40 samples feature nanorods with surfaces decorated by ZnFe_2_O_4_ nanoparticles, resulting in a rougher texture. Specifically, the ZF20 sample, which underwent a shorter immersion period, contains relatively thin (~15 nm) ZnFe_2_O_4_ nanoparticles that do not form a continuous coating, leaving multiple exposed ZnO-ZnFe_2_O_4_ interfaces to the air. However, in ZF40, prolonged immersion leads to a substantial increase in nanoparticle thickness (~28 nm) and the formation of a continuous coating, which likely inhibits interaction between the ZnO-ZnFe_2_O_4_ interface and the surrounding gas environment. Figure 4b,d,f provide detailed insights into the lattice structures of ZF0, ZF20, and ZF40, respectively. In Figure 4b, the tip of a ZnO nanorod is observed, with lattice fringes measuring 0.261 nm, 0.147 nm, and 0.169 nm, corresponding to the (002), (103), and (110) planes of ZnO, respectively. These observations indicate that ZnO predominantly grows along the [002] direction, with the (110) plane exposed on the lateral surfaces. Additionally, a needle-like structure formed at the tip of the ZnO nanorod, exposing the (103) crystal plane. Wang et al. prepared [002]-oriented ZnO thin films using the RF sputtering method with the (103) crystal plane observed on the film surface, which is very similar to the ZnO we synthesized. The appearance of the (103) plane may be related to atomic movement or diffusion during the final stage of the reaction [35]. We hypothesize that the formation of the (103) plane could contribute to the reduction of the system’s energy, as the surface energy of the (002) plane is higher than that of the (103) plane [36]. In Figure 4d, lattice fringes of 0.300 nm and 0.264 nm within the nanoparticles are attributed to the (220) and (310) planes of ZnFe_2_O_4_, respectively, while the 0.165 nm lattice fringe observed in the nanorods corresponds to the (110) plane of ZnO. The ZnFe_2_O_4_ nanoparticles are observed to adhere tightly to the nanorods, forming ZnFe_2_O_4_-ZnO heterojunctions. Figure 4f displays lattice fringes of 0.298 nm and 0.373 nm within the nanoparticles, corresponding to the (220) and (210) planes of ZnFe_2_O_4_, respectively, while the 0.260 nm fringe in the nanorod corresponds to the (002) plane of ZnO. The clear lattice fringes at the interface further confirm the formation of the ZnFe_2_O_4_-ZnO heterojunction.

**Figure 5:**
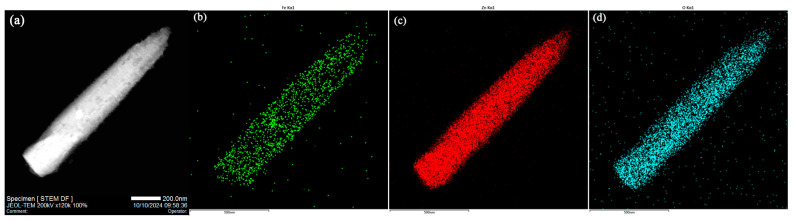
(**a**) The TEM image of the ZF20 material and the EDS mapping images for (**b**) Fe, (**c**) Zn, and (**d**) O elements.

Figure 5 presents the EDS mapping results for the ZF20 sample, revealing a uniform distribution of Zn and O elements throughout the nanorods. In contrast, the Fe content is relatively lower compared to Zn, but it is evenly distributed on the surface of the nanorods.

**Figure 6:**
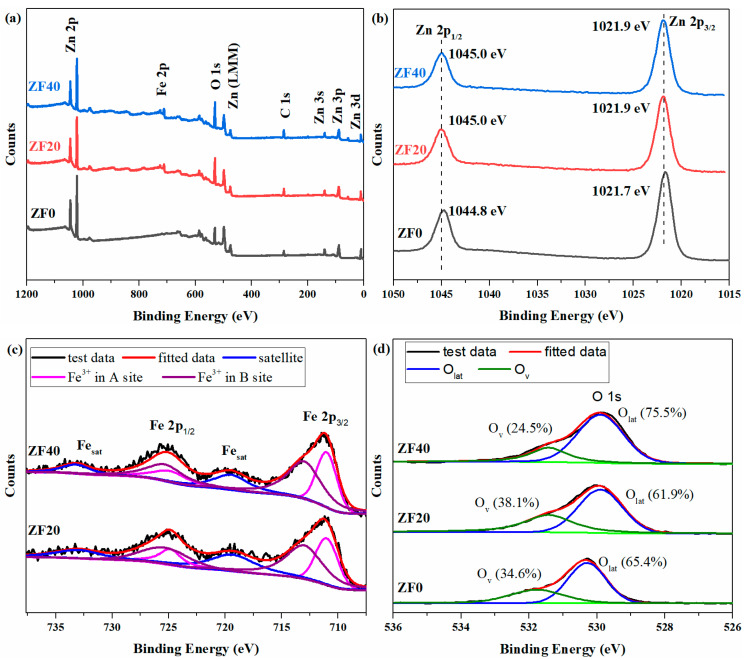
XPS spectra of ZF0, ZF20, and ZF40 for (**a**) full-survey, (**b**) Zn 2p, (**c**) Fe 2p, and (**d**) O 1s.

XPS was employed to analyze the elemental composition and valence states of the materials. The C 1s peak of all samples is calibrated to 284.8 eV. Figure 6a shows the survey spectra, where Zn, O, and C elements are detected in all samples, while Fe is only observed in the ZF20 and ZF40 samples. Figure 6b presents the Zn 2p spectra for the three samples. The peaks at approximately 1044.8 eV and 1021.7 eV correspond to the Zn 2p1/2 and Zn 2p3/2 spin-orbit levels, respectively [37]. The Zn 2p binding energies of ZF20 and ZF40 are shifted 0.2 eV higher than those of ZF0, which may be attributed to two factors. (1) ZnFe_2_O_4_ nanoparticles grow on the surface of ZnO nanorods, and the binding energy of Zn 2p in ZnFe_2_O_4_ [38] differs from that in ZnO [39]. A similar phenomenon, where the Zn 2p binding energy shifts to higher energies in ZnFe_2_O_4_, has been reported in other studies as well [40,41]. (2) The ZnO-ZnFe_2_O_4_ heterojunction is formed. As suggested by other researchers, changes in the distribution of space charge and band bending at the heterojunction interface can lead to the shift in the binding energy [42,43,44]. Ma et al. proposed that localization, densification, entrapment, or polarization of charge, energy, and mass occur at the heterojunction. The energy stored at the interface perturbs the Hamiltonian, resulting in core level shifts [45]. Figure 6c shows the Fe 2p spectra for the ZF20 and ZF40 samples, which can be deconvoluted into six peaks [20,37]. The Fe 2p3/2 peak is split into two peaks at 711.0 eV and 713.0 eV, while the Fe 2p1/2 peak is split into two peaks at 724.9 eV and 725.3 eV, corresponding to Fe^3^^+^ at the A site (tetrahedral lattice point) and B site (octahedral lattice point) of the spinel structure, respectively. The broad peaks at 719.4 eV and 733.2 eV are identified as satellite peaks of Fe 2p orbitals. Figure 6(d) presents the O 1s spectra for the samples, which can be deconvoluted into two peaks, representing lattice oxygen (O_lat_) and oxygen vacancies (O_v_). In ZF0, the O_lat_ and O_v_ peaks are located at 530.3 eV and 531.7 eV, respectively [37], while in ZF20 and ZF40, they are shifted to 529.9 eV and 531.4 eV due to the existence of ZnFe_2_O_4_. The relative proportions of lattice oxygen and oxygen vacancies are calculated from the peak areas and labeled in Figure 6d. The oxygen vacancy content in ZF20 and ZF40 exceeds that of ZF0, with ZF20 having the highest oxygen content, reaching 38.1%. This can be attributed to the material’s synthesis process. Research has demonstrated that Fe_2_O_3_, obtained through the high-temperature decomposition of FeOOH in air, exhibits a high oxygen vacancy content, reaching approximately 40% [29,46]. According to Equation (4), the Fe_2_O_3_ used in the material synthesis process is also derived from the thermal decomposition of FeOOH in air, indicating that the Fe_2_O_3_ itself inherently contains a high density of oxygen vacancies. This characteristic likely contributes to the higher oxygen vacancy content in ZF20 and ZF40, which are synthesized from Fe_2_O_3_, as shown in Equation (5). TEM analysis reveals that ZF20 exhibits thinner and more discontinuous ZnFe_2_O_4_ layers, along with smaller ZnFe_2_O_4_ particles. Additionally, the broader diffraction peaks of ZnFe_2_O_4_ observed for ZF20 suggest a smaller particle size than ZF40. Smaller particles typically exhibit higher surface energy and a greater number of defects [37,47]. During the synthesis of ZnFe_2_O_4_, atomic diffusion and phase transition-induced internal stress may also contribute to the formation of oxygen vacancies [44], with the effect of stress being more pronounced in smaller particles. Therefore, the smaller particle size of ZnFe_2_O_4_ in ZF20 explains its higher concentration of oxygen vacancies.

Oxygen vacancies are crucial for gas sensors, as the gas-sensing response arises from chemical reactions and charge transfer processes involving adsorbed oxygen, and oxygen vacancies serve as effective sites for oxygen adsorption [48]. An increase in oxygen vacancies enhances oxygen adsorption on the surface, thereby improving the response.

### 3.2. Gas Sensing Properties

**Figure 7:**
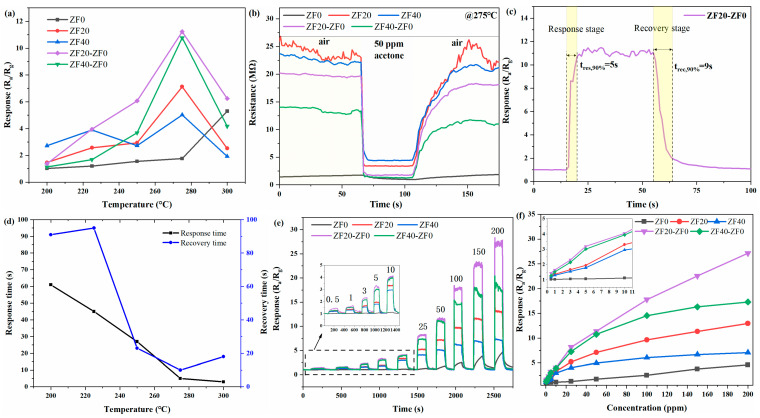
(**a**) Temperature dependence of sensor responses to 50 ppm acetone. (**b**) Resistance curves at 275 °C for all sensors exposed to 50 ppm acetone. (**c**) Response curve of the ZF20-ZF0 sensor to 50 ppm acetone at 275 °C. (**d**) Response and recovery times of the ZF20-ZF0 sensor to 50 ppm acetone at different temperatures. (**e**) Response curves of all sensors to acetone concentrations ranging from 0.5 to 200 ppm at 275 °C and (**f**) the corresponding concentration dependence curves.

Three single-layer gas sensors were first fabricated using the prepared ZF0, ZF20, and ZF40 materials. Then, inspired by reports in the literature suggesting that bilayer structures can enhance gas response and selectivity [23,24,25], two bilayer gas sensors were also fabricated. These bilayer sensors consist of a ZF20 or ZF40 layer as the base, with a ZF0 layer as the top covering. Comparative experiments were conducted on these gas sensors to analyze their temperature dependence and response-recovery characteristics, as shown in Figure 7.

Reliable heating elements are typically integrated into semiconductor gas sensors, as gas-sensitive materials often require high temperatures to exhibit optimal chemical activity and gas response. Figure 7a illustrates the sensor response to 50 ppm acetone within a working temperature range of 200 °C to 300 °C. The response of ZF0 increases with temperature, reaching a maximum of 300°C. In contrast, the other four sensors show their peak response at 275 °C, indicating that the introduction of ZnFe_2_O_4_ successfully lowered the operational temperature of the sensors. The increase in temperature impacts the gas sensor in three primary ways [20,49]. (1) It facilitates redox reactions between reducing gases and oxygen ions on the surface of the semiconductor. (2) It increases the number of thermally excited charge carriers within the material. (3) It enables gas molecules to more easily overcome the binding forces of the semiconductor material and flow away. While Way (1) enhances gas sensitivity, Ways (2) and (3) tend to reduce it. For ZF0, Way (1) remains dominant as the temperature rises from 200 °C to 300 °C. For the other four sensors, Way (1) predominates up to 275 °C, but above this temperature, Ways (2) and (3) become dominant. At 275 °C, the sensor responses to 50 ppm acetone follow the order: ZF20-ZF0 > ZF40-ZF0 > ZF20 > ZF40 > ZF0, with corresponding response values of 11.2, 10.8, 7.1, 5.0, and 1.8. The response of ZF20 is 3.9 times higher than that of ZF0, indicating that the modification of ZnO nanorods with ZnFe_2_O_4_ nanoparticles not only lowers the working temperature but also enhances gas sensitivity. Furthermore, the response of ZF20-ZF0 is 1.6 times that of ZF20, demonstrating that the bilayer structure further amplifies the gas-sensing response.

Figure 7b illustrates the resistance variation of five sensors exposed to 50 ppm acetone at 275 °C. Upon introducing acetone into the test chamber, all sensors displayed a decrease in resistance. The ZF0 sensor showed a gradual reduction over 40 s, indicating a slower response, while the other four sensors reached their response levels within 6 s. Upon re-exposure to air, each sensor’s resistance returned close to its initial value, demonstrating effective recovery. The baseline resistance of the five sensors in air followed the order ZF20 > ZF40 > ZF20-ZF0 > ZF40-ZF0 > ZF0, with corresponding resistance values of 23.3 MΩ, 22.1 MΩ, 19.6 MΩ, 13.5 MΩ, and 1.8 MΩ, respectively. The modification of ZnO nanorods with ZnFe_2_O_4_ nanoparticles resulted in an order-of-magnitude increase in resistance, likely due to the formation of heterojunctions between ZnO and ZnFe_2_O_4_, which create a depletion layer at the ZnO-ZnFe_2_O_4_ interface and contribute to the increased sensor resistance [19]. In bilayer sensors, the addition of a ZnO top layer reduced the resistance. However, it still remained over 7.5 times greater than that of the ZF0 sensor. We hypothesize that heterojunctions and potential barriers form between the ZnO nanorods top layer and the ZnFe_2_O_4_ nanoparticles bottom layer. This potential barrier diminishes the influence of the ZnO top layer, thereby preserving the dominance of the bottom layer’s resistance.

Figure 7c illustrates the response variation curve of the ZF20-ZF0 bilayer gas sensor exposed to 50 ppm acetone, with marked regions indicating the response and recovery times. The sensor exhibits rapid response capabilities, achieving response and recovery times of 5 s and 9 s, respectively. Figure 7d further shows the response and recovery times of the ZF20-ZF0 sensor at different temperatures for 50 ppm acetone. At temperatures below 250 °C, the sensor’s response and recovery are relatively slow. However, when the temperature exceeds 250 °C, the response and recovery times significantly decrease to within 30 s, highlighting the substantial influence of temperature on the catalytic activity of the semiconductor surface. Additionally, the recovery time at 300 °C is slightly longer than at 275 °C. In calculating the recovery time, we focused on the phase corresponding to 90% of the response change. As seen in Figure 7c, the sensor requires additional time to complete the final 10% of the recovery, during which the rate of response change is relatively slow. The lower response value at 300 °C places the 90% response change interval within this slower recovery phase, resulting in a longer recovery time.

Figure 7e,f depict the response variation curves and the response–concentration relationship of the prepared gas sensors at 275 °C towards various acetone concentrations. The sensors’ response increases with rising acetone concentration, with the ZF20-ZF0 sensor showing a higher response than other sensors. At concentrations below 25 ppm, the response rises rapidly with increasing concentration, whereas above 25 ppm, the rate of response growth becomes slower. In addition, except for ZF0, all other gas sensors can achieve a detection limit as low as 0.5 ppm.

**Figure 8:**
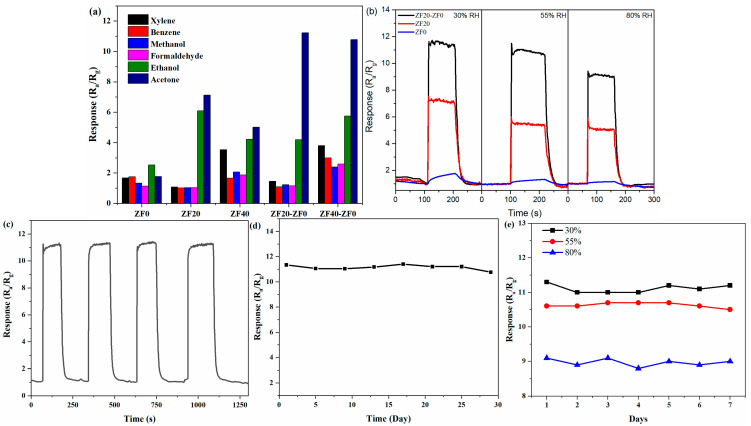
(**a**) Selectivity test of the prepared sensors to 50 ppm different gases at 275 °C. (**b**) The response of the ZF20-ZF0, ZF20, and ZF0 gas sensors to 50 ppm acetone at different humidity. (**c**) Repeatability and (**d**) long-term stability test of the ZF20-ZF0 gas sensor to 50 ppm acetone. (**e**) After being stored in a high-humidity environment, the responses of the ZF20-ZF0 gas sensor to 50 ppm acetone at different humidity levels over one week.

For gas sensors, interfering gases induce additional responses, leading to an overestimation of the sensor’s response to the target gas. Increased humidity often impedes the response of semiconductor gas sensors, resulting in an underestimation of the response to the target gas. In practical applications of gas sensors, it is crucial to account for the influence of interfering gases and humidity on sensor performance. Figure 8a displays the responses of five gas sensors to 50 ppm of acetone, ethanol, formaldehyde, methanol, benzene, and xylene. Unlike other sensors, ZF0 is more sensitive to ethanol gas, owing to the stronger adsorption of ethanol on the unmodified ZnO surface [17]. By contrast, the ZF20 and ZF40 sensors exhibit the strongest response to acetone, suggesting that the ZnFe_2_O_4_ nanoparticles decoration on the ZnO nanorods significantly enhances acetone sensitivity, which is due to ZnFe_2_O_4_’s intrinsic affinity for acetone [18]. Meanwhile, their responses to ethanol are lower but still comparable to those for acetone, indicating potential interference from ethanol with the ZF20 and ZF40 sensors. To address this, an additional ZnO thin film was applied to the sensor surface, which improved the selectivity of ZF20-ZF0 and ZF40-ZF0 sensors. This enhancement is evident from the considerably stronger response to acetone compared to ethanol due to the ZnO layer’s selective filtration effect. ZnO exhibits high adsorption and catalytic activity for ethanol, so the ethanol molecules may be adsorbed or oxidized as they pass through the top layer, thereby diminishing ethanol’s interference with the bottom sensing layer.

Figure 8b shows the response curves of the ZF20-ZF0, ZF20, and ZF0 sensors to 50 ppm acetone under varying humidity levels. All sensors exhibit a gradual decline in response as humidity increases. At 30%, 55%, and 80% RH, the responses are 11.4, 10.7, and 9 for ZF20-ZF0, 7.1, 5.4, and 5.1 for ZF20, and 1.8, 1.3, and 1.2 for ZF0, respectively. From 30% RH to 80% RH, the responses of ZF20-ZF0, ZF20, and ZF0 decrease by 21%, 28%, and 33%, respectively. The humidity resistance can be ranked in the following order: ZF20-ZF0 > ZF20 > ZF0.

Since gas sensors operate at elevated temperatures, it is crucial to understand the behavior of water on material surfaces at different temperatures. Raymond et al. conducted molecular dynamics simulations using the ReaxFF force field to investigate the adsorption behavior of water molecules on ZnO surfaces at various temperatures. At room temperature, water molecules can achieve 100% coverage of the ZnO surface, with half of the water molecules dissociating. At elevated temperatures around 300 °C, the increase in temperature promotes gas desorption, reducing water coverage on ZnO to 60%. On the flat plane, approximately half of the water molecules still dissociate, while on the stepped plane, dissociation is more pronounced [50]. Iachella et al. used DFT simulations to explore the adsorption properties of water on various ZnO surfaces. At room temperature, water molecules spontaneously dissociate on all surfaces except the O-terminated polar surface. The dissociated protons can bond with either oxygen or zinc atoms. The OH group can achieve up to 1/4 ML coverage on the surface, and defects such as oxygen vacancies further enhance coverage. Increasing the temperature reduces water coverage but promotes a higher degree of dissociation. At 400 °C, OH remains readily adsorbed on the surface, while complete desorption occurs above 900 °C [51]. These findings suggest that, around the operating temperature of gas sensors (275 °C), water molecules will adsorb significantly on the sensor surface, and the dissociation of water should not be overlooked. The above conclusions help us analyze the impact of humidity on gas sensors. We attribute the response decay caused by humidity to two main reasons: (1) adsorbed water molecules may adsorb on ZnO surface [52,53], occupying sites that would otherwise be available for oxygen or acetone, hindering their adsorption [20]; (2) water molecules can react with adsorbed oxygen ions to form -OH groups, thereby reducing the quantity of reactive oxygen ions and subsequently decreasing the sensor’s response [54,55].

The repeatability and long-term stability of a sensor are critical for ensuring data reliability, which is essential for practical applications. Figure 8c shows the response curves of the ZF20-ZF0 bilayer sensor during four consecutive tests of 50 ppm acetone at 275 °C. The response values remain highly consistent across each test, indicating reproducible performance. Figure 8d shows the response values of the ZF20-ZF0 sensor to 50 ppm acetone over a 29-day testing period at 275 °C, during which no significant decrease in response is observed. These results confirm the ZF20-ZF0 sensor’s excellent repeatability and long-term stability. Furthermore, following long-term stability testing, the ZF20-ZF0 sensor was placed in an 80% RH environment for five days, after which it underwent another week of testing under varying humidity conditions. The results are shown in Figure 8e. Even after being stored in a high-humidity environment, the sensor exhibited remarkable stability in its response across different humidity levels.

In Table 2, we compared the performance of the ZF20-ZF0 bilayer gas sensor with other acetone gas sensors reported in the literature, including ZnFe_2_O_4_-ZnO composites and other materials. Compared to most sensors listed in the table, our sensor exhibits excellent gas-sensing response and detection limits. PdO-ZnFe_2_O_4_ macroporous spheres and Au-ZnFe_2_O_4_-ZnO microspheres show slightly higher response values due to the enhancement effect of the noble metals. Core-shell ZnO/ZnFe_2_O_4_ spheres and ZnFe_2_O_4_ nanoparticles on ZIF-8-derived ZnO demonstrate ultra-high gas-sensing responses, thanks to the complex structural designs at the micron and nanometer scales. However, these sensors suffer from slow response/recovery speeds, and their resistance in air is 800 MΩ and 2.8 GΩ, respectively. Such high resistance values make them difficult to apply in practical scenarios. In contrast, our sensor has a resistance of around 20 MΩ, which makes it easily detectable using portable devices.

The ZF20-ZF0 bilayer gas sensor developed in this study meets the detection requirements for acetone gas monitoring in factories and laboratories (TWA: 126 ppm, STEL: 190 ppm), as well as for detecting acetone liquid leaks. The sensor is capable of detecting acetone at concentrations as low as 0.5 ppm, which is below the exhaled gas detection threshold of 1.7 ppm for diabetes patients. This makes it a promising candidate for breath analysis. However, further validation is still needed.

When compared to traditional single-layer gas sensors, the large-scale production of the bilayer gas sensor still presents several challenges. The demands on production are undoubtedly higher because it is crucial to precisely control the manufacturing processes of both the upper and lower layers to ensure consistent sensor performance, which requires high-precision automated equipment. Additionally, bilayer sensors incorporate extra processing steps into production, necessitating careful consideration of ways to minimize time and cost.

### 3.3. Gas Sensing Mechanism

In semiconductor gas sensors, the gas-sensitive mechanism is often explained by the charge transfer process caused by the adsorption–desorption process and redox reaction of reducing gases and oxygen ions. Specifically, on the surface of ZnO nanorods or ZnFe_2_O_4_ nanoparticle-decorated ZnO nanorods, oxygen molecules are first adsorbed. At an operating temperature of around 275 °C, due to their high electron affinity, the adsorbed oxygen species capture electrons from ZnO or ZnFe_2_O_4_, forming ionized oxygen species ($O_{2}^{-}\;,O^{-}\;,O^{2-}$) [48,65]. When the sensor is exposed to an acetone-contained atmosphere, acetone reacts with the adsorbed oxygen ions, producing water and carbon dioxide, as represented by the following chemical equation [63]. During this reaction, the oxygen ions release the electrons they had captured, increasing the electron concentration within the n-type semiconductor material, which subsequently leads to a decrease in resistance.

C_3_H_6_O + 8O^−^ = 3CO_2_ + 3H_2_O + 8e^−^ (8)

Through surface modification of ZnO nanorods with ZnFe_2_O_4_ nanoparticles, the acetone sensing performance of the ZF20 and ZF40 sensors is enhanced, which can be attributed to two factors: chemical sensitization and electronic sensitization.

As a potential sensing material for acetone, ZnFe_2_O_4_ has demonstrated strong acetone adsorption by DFT study [18]. Meanwhile, the ZnFe_2_O_4_-ZnO heterojunction enhances the catalytic activity by increasing oxygen vacancies and facilitating charge separation [66,67]. These chemical sensitization effects contribute to improvement both in response and selectivity.

**Figure 9:**
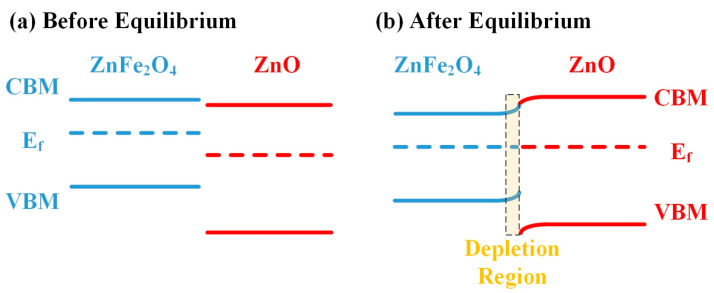
The energy band diagrams of the ZnFe_2_O_4_-ZnO heterojunction (**a**) before equilibrium and (**b**) after equilibrium.

Electronic sensitization refers to the introduction of a depletion layer through heterojunction, increasing the responses [68]. Both ZnFe_2_O_4_ and ZnO are n-type semiconductors. The conduction band minimum (CBM) and valence band maximum (VBM) of ZnFe_2_O_4_ are positioned at −0.2 V and 2.1 V vs. NHE, while for ZnO, the CBM and VBM values are −0.1 V and 3.47 V vs. NHE. A type II heterojunction is formed between ZnFe_2_O_4_ and ZnO since the CBM and VBM of ZnO are both more positive than those of ZnFe_2_O_4_ [20,21,22,69]. Additionally, with the work function of ZnFe_2_O_4_ (4.5 eV [70]) being lower than that of ZnO (5.0~5.3 eV [71,72,73]), electron flow from ZnFe_2_O_4_ to ZnO occurs until Fermi level equilibrium is achieved. The energy band diagram of the ZnFe_2_O_4_-ZnO heterojunction is shown in Figure 9.

As electrons transfer from ZnFe_2_O_4_ to ZnO, the energy bands of ZnFe_2_O_4_ and ZnO bend upward and downward at the interface, respectively, forming a potential barrier. At the interface on the ZnFe_2_O_4_ side, the upward bending of the bands makes the Fermi level shift toward the valence band. As the Fermi level approaches the band gap center, it indicates that the electron and hole concentrations are nearly equal, leading to the formation of a depletion layer on the ZnFe_2_O_4_ side. The band gap center of ZnFe_2_O_4_ is approximately 0.95 eV below the CBM [74]. Since ZnFe_2_O_4_ is undoped, the difference between its Fermi level and the band gap center should be much smaller than 0.95 eV. The work function difference between ZnFe_2_O_4_ and ZnO ranges from 0.5 to 0.8 eV, which equals to the extent of the band bending or the Fermi level downward shift. Additionally, the adsorption of oxygen on the material surface further lowers the Fermi level. Therefore, after equilibrium is reached, the Fermi level of ZnFe_2_O_4_ at the interface is likely to approach the band gap center, resulting in the formation of a depletion layer. Li et al. fabricated ZnFe_2_O_4_/ZnO hollow sphere materials and observed a transition from n-type to p-type conductivity at 300 °C. This demonstrates that the n-n heterojunction of ZnFe_2_O_4_/ZnO can form not only a depletion layer on the ZnFe_2_O_4_ side but also potentially an inversion layer [69].

In p-n heterojunction studies, depletion layers form on both sides of the interface. It has been proposed that nanoparticles can leverage these depletion layers to control the width of the conductive pathway in nanorods or nanosheets [75,76], thereby influencing the sensor response. However, our study presents a different scenario. At the n–n heterojunction interface, a depletion layer forms on the ZnFe_2_O_4_ side, while an accumulation layer forms on the ZnO side due to the influx of electrons. The accumulation layer on the ZnO side does not significantly modulate the conductive pathway of the ZnO nanorods, and its effect on the sensor response is minimal. Therefore, it is crucial to focus on how the depletion layer on the ZnFe_2_O_4_ side influences the response. In this study, ZnFe_2_O_4_ nanoparticles cover a substantial portion of the ZnO surface. As electrons transfer from one ZnO nanorod to another, they must pass through the ZnFe_2_O_4_ nanoparticles. In other words, the depletion layer is positioned along the conduction path, playing a pivotal role in the resistance of the gas sensor. When acetone reacts with the adsorbed oxygen on the material surface, electrons are released back into the conduction bands of ZnFe_2_O_4_ and ZnO, leading to an increase in the electron concentration, a rise in the Fermi level, a reduction in the depletion layer thickness and the potential barrier [77]. The gas sensing response is given by *R*_a_/*R*_g_. The depletion layer has a high response since its *R*_a_ is very high [78,79,80,81]. Therefore, increasing the proportion of the depletion layer in the conductive channel is favorable for enhancing the sensor’s response.

ZF20 exhibits higher sensitivity to acetone compared to ZF40. XPS analysis reveals that ZF20 possesses a higher proportion of oxygen vacancies, which act as adsorption sites for oxygen [48]. With more oxygen adsorbed, both the redox reactions and the gas-sensing response are enhanced. Additionally, as shown in the HRTEM images, the ZnFe_2_O_4_ nanoparticles on the ZnO nanorods in ZF20 are thinner than those in ZF40, thereby allowing the gas-sensing reaction to more effectively modulate the electron distribution within the heterojunction, further boosting gas-sensing performance [82,83,84].

A bilayer structure is fabricated by depositing a layer of ZnO nanorods atop the ZnFe_2_O_4_ nanoparticle-decorated ZnO nanorods, demonstrating an even greater improvement in acetone response and selectivity. This enhancement can be attributed to three key factors. (1) Additional n–n heterojunctions are formed between ZnO nanorods in the top layer and the underlying ZnFe_2_O_4_ nanoparticles in the bottom layer, further increasing the sensitivity of the structure. (2) In the top layer of ZnO nanorods, abundant porosity allows gas molecules to rapidly diffuse into the underlying material. The ZnO nanorods themselves also provide a partial blocking effect. Specifically, acetone molecules that do not react in the bottom layer can collide with the ZnO nanorods in the top layer and be redirected back to the surface of the bottom layer, thereby increasing the likelihood of acetone molecules participating in the reaction. This mechanism is similar to that of hollow-sphere gas-sensing materials, where gas molecules are trapped within the shell, undergoing multiple collisions with the shell surface, which enhances the gas-sensing response [85,86,87]. (3) One advantage of the bilayer structure gas sensor is its ability to enhance selectivity through the filtering effect of the top layer [24]. In this study, the ZnO nanorod top layer acts as a filter for ethanol. Gas-sensing tests demonstrate that ZnO nanorods exhibit a stronger response to ethanol, and simulation calculations confirm their high ethanol adsorption capacity [88,89,90]. As a result, ethanol molecules are predominantly adsorbed upon traversing the ZnO layer, which decreases the underlying material’s response to ethanol and enhances the sensor’s selectivity for acetone.

The test results regarding the effects of humidity show that the sensors’ resistance to humidity follows the order: ZF20-ZF0 > ZF20 > ZF0. The higher performance of ZF20 compared to ZF0 suggests that ZnO is more sensitive to humidity than ZnFe_2_O_4_, which also confirms ZnO’s strong adsorption capacity for water molecules [52,53]. The superior humidity resistance of ZF20-ZF0 may be attributed to a filtering effect similar to that observed for ethanol, where the upper ZnO layer also acts to filter water molecules, thereby reducing the interference caused by humidity.

The material’s porous structure also plays a crucial role in gas-sensing performance [91,92]. If the material undergoes significant agglomeration, causing many surfaces to be covered, or if the pore size is smaller than the gas molecule size, the surface available for gas adsorption becomes severely limited. When the pore size allows gas molecules to enter and is similar to their size, the gas interacts continuously with the pore walls as it diffuses deeper, which slows down its rate of reaching the bottom of the pores. This, in turn, results in a slower sensor response. Conversely, if the pores are large enough, the diffusion of gas within the pores is less hindered by the walls, enabling the gas to diffuse more rapidly to reach equilibrium. Additionally, the abundance of pores increases the surface area in contact with the gas, thereby improving both gas adsorption efficiency and sensor detection speed. As shown in the SEM images, a porous urchin structure forms by the ZnO nanorods, with pores significantly larger than the gas molecules. After modification with ZnFe_2_O_4_ nanoparticles, the porous characteristics are preserved, indicating that this structure provides many adsorption sites and diffusion pathways for the gas. However, the sensor’s detection speed is influenced not only by the material’s morphology but also by factors such as surface adsorption/desorption rates and chemical catalytic activity. Quantifying the contribution of material structure to sensor performance still remains a challenge.

## 4. Conclusions

In this work, ZnO nanorods (ZF0) with a sea-urchin-like morphology were initially synthesized and subsequently immersed in FeCl_3_ solution for 20 or 40 s, followed by calcination at 450 °C to prepare ZnFe_2_O_4_ nanoparticle-decorated ZnO nanorods (ZF20 and ZF40). The microstructure, morphology, and elemental composition of the materials were analyzed using XRD, SEM, TEM, EDS, and XPS. Three single-layer gas sensors were fabricated. ZF20 demonstrated the best performance, showing a response to acetone that was 3.9 times higher than pure ZnO, while also achieving a reduced operating temperature. This enhancement is attributed to ZF20’s porous microstructure, abundant oxygen vacancies for oxygen adsorption, and extensive ZnO-ZnFe_2_O_4_ heterojunctions. In ZF20, the thin layer of ZnFe_2_O_4_ particles on the ZnO surface promotes gas modulation of the depletion layer thickness within the heterojunctions. Two bilayer gas sensors (ZF20-ZF0 and ZF40-ZF0) were further prepared by covering ZF20 and ZF40 with an additional ZnO nanorod layer. This bilayer configuration not only increased the acetone response but also significantly improved acetone selectivity. The enhanced performance can be ascribed to resistance modulation from the heterojunctions between the two layers, partial blocking of unreacted acetone gas and filtering of interfering ethanol molecules by the ZnO top layer. Moreover, the bilayer structure sensor exhibited quick response/recovery times (5/9 s), excellent repeatability, and long-term stability. This study provides valuable new insights for designing high-performance gas sensors.

## Figures and Tables

**Table 1:** The structural parameters and lattice strain of the samples.

Sample	Lattice Parameters			d_(101)_ (Å)	Lattice Strain (%)
	a (Å)	b (Å)	c (Å)		
ZF0	3.25445	3.25445	5.21044	2.4790	0.193
ZF20	3.24835	3.24835	5.20017	2.4743	0.217
ZF40	3.25037	3.25037	5.20315	2.4758	0.207

**Table 2:** Acetone gas sensing performance of ZnFe_2_O_4_-ZnO composites and other materials.

Materials	*T* (°C)	*C* (ppm)	*S* (Ra/Rg)	LOD (ppm)	*t*_res_/*t*_rec_ (s)	Ref
TiO2 nanoparticle	270	1000	15.4	0.5	10/9	[56]
TiO2 nanorod	320	100	12.3	1	3/421	[57]
Sn doped ZnO nanorod	300	200	6.3	5	7/32	[58]
ZnFe_2_O_4_ microspheres	279	100	8.4	1.7	4/5	[59]
ZnFe_2_O_4_ macroporous sphere	275	100	9	5	7/67	[60]
PdO-ZnFe_2_O_4_ macroporous sphere	275	100	18.9	5	5/54	[60]
ZnFe_2_O_4_-ZnO flower	250	100	9.7	1	2/25	[61]
ZnFe_2_O_4_-ZnO hollow sphere	280	100	5.7	10	6.6/7.5	[62]
double shell ZnFe_2_O_4_-ZnO hollow sphere	250	100	16.8	5	1/33	[63]
triple shell ZnFe_2_O_4_-ZnO hollow sphere	140	100	14	5	5.2/12.8	[64]
ZnFe_2_O_4_-ZnO microspheres	279	100	11.6	1.2	4/8	[59]
Au-ZnFe_2_O_4_-ZnO microspheres	206	100	18.18	0.7	4/23	[59]
Core–shell ZnO/ZnFe_2_O_4_ spheres	250	100	94	0.05	56/89	[19]
ZnFe_2_O_4_ nanoparticles on ZIF-8-derived ZnO	260	100	225	0.05	6/158	[20]
ZnO/ZnFe_2_O_4_-ZnO bilayer	275	100	17.8	0.5	5/9	This work

*T*, Operating temperature. *C*, acetone concentration. *S*, response. LOD, limit of detection. *t*_res_, response time. *t*_rec_, recovery time. Ref, reference.

## Data Availability

The data will be made available upon request.
